# *Klebsiella pneumoniae*-related invasive liver abscess syndrome complicated by purulent meningitis: a review of the literature and description of three cases

**DOI:** 10.1186/s12879-020-05702-3

**Published:** 2021-01-06

**Authors:** Ruixue Sun, Hui Zhang, Yingchun Xu, Huadong Zhu, Xuezhong Yu, Jun Xu

**Affiliations:** 1 Emergency Department, Peking Union Medical College Hospital, No.1 shuaifuyuan, Dongcheng District, Beijing, China; 2 Laboratory Department, Peking Union Medical College Hospital, No.1 shuaifuyuan, Dongcheng District, Beijing, China

**Keywords:** *Klebsiella pneumoniae*, Meningitis, Liver abscess, Case report

## Abstract

**Background:**

*Klebsiella pneumoniae* (*K. pneumoniae*) invasive liver abscess syndrome (ILAS) with purulent meningitis was rarely identified the mainland of China. Last winter, we received 3 cases of *K. pneumoniae* meningitis and all of them died in a short time. We report these cases in order to find the reason of high mortality and discuss effective effort to improve these patients’ prognosis.

**Case presentation:**

Three patients with uncontrolled diabetes developed live abscess and purulent meningitis. Upon admission, the clinical manifestations, laboratory result of blood and cerebrospinal fluid (CSF) and imaging examinations were compatible with *K. pneumoniae* ILAS which had metastasis infection of meningitis. Even with timely adequate antibiotic therapy and strict glycemic control, all of the patients’ condition deteriorated rapidly and died in a short time.

**Conclusion:**

The reason of patients’ poor prognosis might be the absence of liver abscess drainage, high level of CSF protein which indicates severe inflammation and unknown special but stronger virulence factors of *K. pneumoniae* the patients’ living place Zhangjiakou. Strict glycemic control, early drainage of liver abscess and appropriate antibiotic application are recommended for treating this condition, further progress on the pathogenesis and treatment of *K. pneumoniae* meningitis may help patients gain a better prognosis.

## Background

Over a century ago, bacterial meningitis was virtually 100% fatal [1]. Despite current antibiotics being able to clear bacteria from the cerebrospinal fluid (CSF), mortality remains approximately 25%, and, even among survivors, 21–28% of patients have chronic neurologic complications [2, 3]. *Streptococcus pneumoniae* is the most common bacterial cause of community-acquired meningitis in adults. The distribution of other pathogens, such as *Neisseria meningitidis*, *group B Streptococcus, Haemophilus influenzae* and *Listeria monocytogenes*, depends upon the age of the patient, their vaccination status and the regional epidemiological trends where the patient lives [4, 5]. Besides the more common organisms listed above, *Klebsiella pneumoniae (K. pneumoniae)* has been reported as a cause of community-acquired meningitis in Taiwan with a mortality rate of 30–40% in patients with liver abscesses [6, 7]. Diabetes and age > 65 years old were independent predictors of septic ocular or CNS complications in patients with liver abscesses [8]. Besides Taiwan, in South Korea, *K. pneumoniae* was the third most common cause of community-acquired bacterial meningitis [9]. However, *K. pneumoniae* meningitis is rarely identified in other regions, including mainland China. In 2018, our emergency department received three cases of *K. pneumoniae* meningitis. These three initially presented with fever and altered mental status, and all three died within two days after admission. We report these cases in order to illustrate the early signs of *K. pneumoniae* meningitis and discuss potentially effective strategies to improve the prognosis of these patients.

## Case presentations

### Case 1

At 07:23 on June 3, 2018, we received a 39-year-old male patient in our emergency room. He came from Zhangjiakou in Hebei province, China and presented with five days of fever and three hours of altered mental status. He had a history of poorly controlled diabetes mellitus. At the time of admission, his vital signs included a body temperature of 39 °C, a heart rate of 146 beats/min, blood pressure of 125/84 mmHg, respiratory rate of 42 breaths/min, and an oxygen saturation of 99% on room air. He had a Glasgow coma scale (GCS) of E1 + V1 + M3 with nuchal rigidity on an otherwise unremarkable physical examination.

**Fig. 1:**
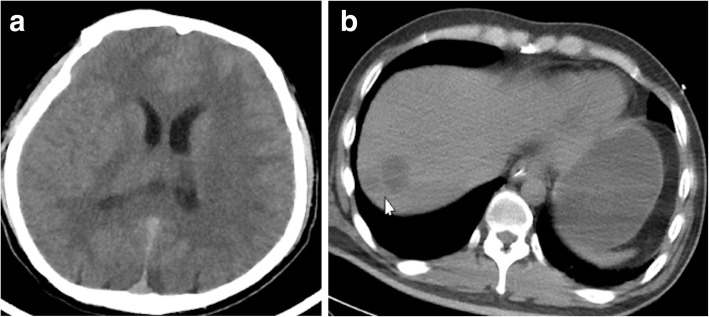
**a**: Head CT scan showed diffuse cerebral edema; **b**: Abdominal CT scan demonstrating an area of abnormal attenuation measuring 24 mm × 19 mm in the right lobe of the liver, suggestive of a single abscess

White blood cell count was 25.73 × 10^9^/L with an elevated neutrophil percentage of 84.9%. The concentration of C-reactive protein (CRP) was > 240 mg/L, glucose was 30 mmol/L, arterial blood gas (ABG) results were as follows (on 3 L/min of oxygen via nasal cannula): pH: 7.09, arterial pressure of CO_2_ (PaCO_2_): 9.7 mmHg, arterial pressure of oxygen (PaO_2_): 141 mmHg, HCO_3_: 2.8 mmol/L, lactic acid: 3.9 mmol/L, liver and renal function tests were within normal limits. Alb:39 g/L, PCT:2-10 ng/ml. Two sets of peripheral blood cultures were obtained (and were negative). Head and abdominal computed tomography (CT) scans demonstrated diffuse cerebral edema and possible brain and liver abscesses (see Fig. 1).

The patient next underwent a lumbar puncture examination. The opening pressure was 150 mm H_2_O. CSF appeared yellow and purulent, revealing a 178,640 × 10^6^/μL white blood cell count with a multinucleated cells percentage of 86.2%, protein 32.41 g/L and glucose 10.2 mmol/L. CSF was submitted for Gram staining and bacterial culture. The above findings led to the diagnoses of purulent meningitis, sepsis, diabetic ketoacidosis (DKA), as well as (possible) brain and liver abscesses. Meropenem 2 g every 8 h combined with vancomycin 1 g every 12 h were given intravenously. Due to the loss of spontaneous breathing, mechanical ventilation was started. Intensive care was begun and his DKA was treated. However, further invasive examinations (e.g. brain or liver abscess aspirations) were deemed too dangerous for him. At 11:41, the patient was transferred to the Emergency Department’s Intensive Care Unit (EICU).

By June 4, the patient’s condition had not improved. His body temperature fluctuated between 35.3 °C and 39.5 °C. His blood pressure dropped to 91/64 mmHg while the pulse rate rose to 142 beats/min. Norepinephrine was pumped venously to maintain a mean arterial pressure (MAP) > 65 mmHg. Pupils were bilaterally dilated at 3 mm’s with a slow light reflex, a GCS score of E1 + (Ventilated) + M1. Based on his worsening situation, emergency bedside lateral ventricular drainage was performed. During the operation, his cerebrospinal fluid was examined again, demonstrating a white blood cell count of 19,054 cells/μL with a multinucleated cells ratio of 78.9%, protein of 1.06 g/L and a glucose level of 0.1 mmol/L. After the operation, his intracranial pressure was consistently over 330mmH_2_O while undergoing continuous renal replacement therapy along with a maximal dose of mannitol.

*K. pneumoniae* was isolated from the patient’s cerebrospinal fluid and was found to be sensitive to all tested antibiotics including meropenem. However, the patient’s vital signs deteriorated, and he eventually died of cardiac arrest later on June 4.

### Case 2

A 49-year-old woman was admitted to the emergency department of our hospital at 14:00 on July 3, 2018 because of fever and “twitching” for the past three days. She was born and currently lived in Zhangjiakou city of Hebei Province, she had a history of untreated diabetes mellitus and hypertension. At the time of admission, the patient was found to have intermittent convulsions and was unable to cooperate for a physical examination. Her temperature was 38 °C, pulse rate was 152 beats/min, respiratory rate 39 breaths/min, blood pressure was 158/88 mmHg, and her oxygen saturation was 92% on 3 L/min of oxygen via nasal cannula.

**Fig. 2:**
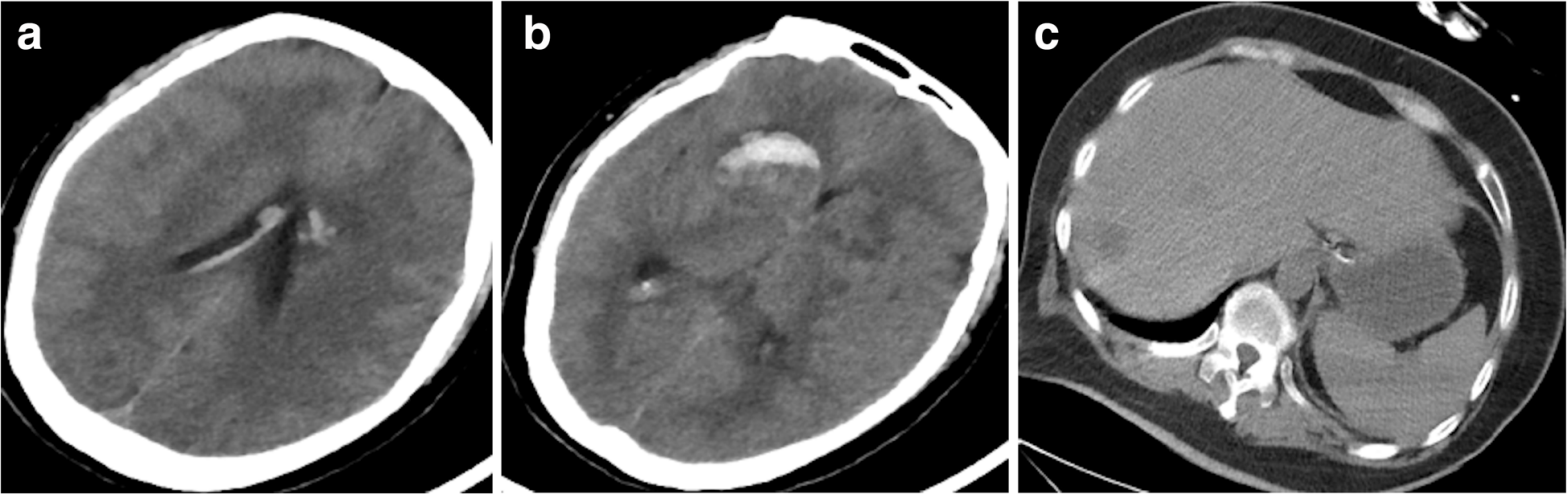
**a and b**: head CT scan showed bilateral basal ganglia hemorrhages and a subarachnoid hemorrhage; **c**: Abdominal CT scan demonstrating an area of abnormal attenuation measuring 18 mm × 14 mm in the right lobe of the liver

The patient’s white blood count was 24.87 × 10^9^/L with an elevated neutrophil percentage of 91.4%, a hemoglobin of 148 g/L and a platelet count of 108 × 10^9^/L. The CRP was 242 mg/L, glucose was 42.4 mmol/L. Arterial blood gas results were as follows (on 3 L/min oxygen via nasal cannula): pH of 7.09, PaCO_2_: 9.7 mmHg, PaO_2_: 141 mmHg, HCO_3_: 2.8 mmol/L, lactic acid: 3.9 mmol/L, creatinine 125 μmol/l, Urea 13.9 mmol/l, other liver and kidney function testing was normal. The patient’s procalcitonin level was > 10 ng/ml. Head CT scans revealed bilateral basal ganglia hemorrhages and a subarachnoid hemorrhage. Abdominal CT scan demonstrated a possible liver abscess (Fig. 2). Puncture examination showed the intracranial pressure was > 330 mm H2O. CSF was pale red and purulent, and revealed 4263 × 10^6^/L white blood cells/μL with multinucleated cells ratio of 43.1%, protein 8.82 g/L and glucose 0.6 mmol/L.

The patient was diagnosed with purulent meningitis, septic shock, cerebral hemorrhage, subarachnoid hemorrhage, DKA and a probable liver abscess. The patient was intubated for airway protection and mechanical ventilation was begun. Meropenem 2 g every 8 h was given intravenously, and lateral ventricular drainage was performed at 12:00. After the operation, the patient’s condition did not improve, and she died that afternoon due to cerebral herniation.

Two days later, blood, sputum and cerebrospinal fluid cultures all grew *K. pneumoniae* (sensitive to all the tested antibiotics including Meropenem).

### Case 3

A 62-year-old male also from Zhangjiakou city of Hebei Province with a history of uncontrolled diabetes mellitus was admitted to the emergency department after he developed confusion for the past 11 h. Two days ago, the patient complained of a fever and vomiting. The patient was seen at a different local hospital, but his condition worsened. On arrival to our hospital, his initial vital signs were: body temperature, 37 °C, heart rate 119 beats/min, blood pressure, 159/126 mmHg, respiratory rate, 32 breaths/min, and oxygen saturation 92% on air. His physical examination was significant for a GCS of 5 (E1 + V1 + M3).

**Fig. 3:**
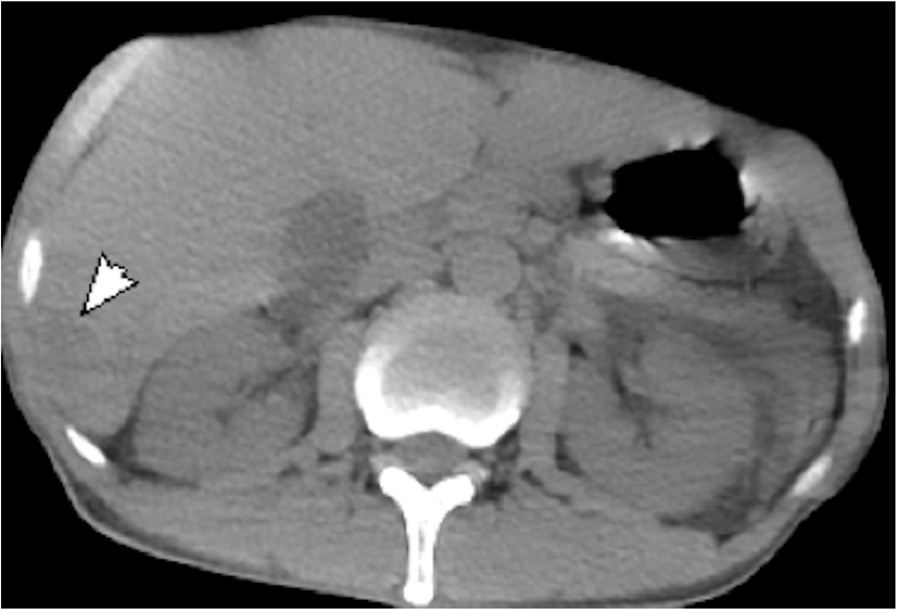
Abdominal CT scan demonstrating an area of abnormal attenuation measuring 25 mm × 19 mm in the right lobe of the liver

His white blood count was 12.39 × 10^9^/L with an elevated neutrophil percentage of 92.8%, hemoglobin 165 g/L and a platelet count 25 × 10^9^/L. ABG results were: pH 7.28, PaCO_2_: 24.1 mmHg, PaO_2_: 89.3 mmHg, HCO_3_: 10.9 mmol/L, lactic acid: 3.4 mmol/L, CRP was > 160 mg/L, procalcitonin > 100 ng/ml, blood glucose was 24.1 mmol/L, alanine transaminase was 139 U/L, total bilirubin/direct bilirubin were 62.0/31.4umol/L, creatinine was 274umol, and urea was 22.79 mmol/L. An abdominal CT scan demonstrated a single liver abscess (Fig. 3). A lumbar puncture was performed after the patient received a platelet transfusion. The opening pressure was too low to measure, and the CSF was a murky yellow and revealed 17,148 × 10^6^/L white blood cells/μL with a multinucleated cells percentage of 84.3%, protein of 12.82 g/L and a glucose of < 0.1 mmol/L. The patient was diagnosed with purulent meningitis, septic shock and DKA. Meropenem 2 g every 8 h combined with vancomycin 1 g every 12 h were given intravenously and blood glucose (BG) was well controlled by insulin. However, the patient got worse and his relatives decided to withdraw care. A telephone follow-up revealed that the patient died on his way back home. Two days later, *K. pneumoniae* was isolated from his CSF and was sensitive to all available antibiotics.

## Discussion and conclusions

Cases of spontaneous *K. pneumoniae* meningitis are rare and commonly observed in hospitalized postoperative patients. Community-acquired *K. pneumoniae* meningitis could be associated with an invasive liver abscess syndrome (ILAS). In a study, two cases (2/15, 13.3%) were associated with *K. pneumoniae* liver abscess. In another study from South Korea, 4 (14.8%) of 27 patients with *K. pneumoniae* meningitis had a concomitant liver abscess [10]. But this situation is still rare, in a study, only 1 (0.9%) of 112 patients with *K. pneumoniae* liver abscess had CNS involvement [11]. We reviewed the case reports and related original articles about adult community-acquired *K. pneumoniae* meningitis and found that only two cases have been recorded in the mainland of China [12, 13]. As all three patients we received died relatively quickly, we summarized above the key points of their diagnosis and treatment and will now shift to analyzing the reasons for our patients’ unfortunate outcomes.

The three patients with community-acquired *K. pneumoniae* meningitis in this article had poorly controlled T2DM. Meningitis was preceded by an ILAS. Accordingly, when patients with T2DM present with fever, headache, coma and confusion, a high degree of suspicion should be held for ILAS besides meningitis, including possible *K. pneumoniae* infection [14]. However, sometimes community-acquired *K. pneumoniae* meningitis appears independently without a liver abscess [13, 15–17]. Besides T2DM, other risk factors may include alcohol-related chronic diseases, especially alcoholic cirrhosis, which is also related to ILAS [15, 16, 18]. The clinical manifestation and high leukocyte, high protein, and low glucose in each patients’ CSF are often found in cases of gram-negative bacilli meningitis [19]. So, the key points for diagnosing community-acquired *K. pneumoniae* meningitis are risk factors and pathogen culture.

Currently, there are no clear guidelines for the management of *K. pneumoniae* meningitis as a manifestation of ILAS. Besides strict glycemic control, a combination of early drainage of the abscess and appropriate antibiotic application is the standard treatment for this condition [20]. The selection of antibiotics should be based on in-vitro susceptibilities and clinical response. In view of the better penetration into the CSF, large doses of third-generation cephalosporins including cefotaxime (up to 2 g every four hours) and ceftriaxone (2 g twice a day) are the drugs of choice for *K. pneumoniae* meningitis. Imipenem and meropenem can be given to patients when strains containing extended-spectrum beta-lactamases are suspected [19]. As reported in the literature, surviving patients with *K. pneumoniae* meningitis were treated with ceftazidime, ceftriaxone, cefmetazole, cefotaxime, cefepime or meropenem [13, 16, 17, 21–27]. In our cases, *K. pneumoniae* cultured from the CSF were susceptible to all the remaining antibiotics tested, but meropenem was the antibiotic chosen for all three patients, and vancomycin was also used in two of them.

Unfortunately, each patient’s ending was still tragic. We noticed that the CSF protein levels in our patients (32.41 g/L, 8.82 g/L and 12.82 g/L, respectively) were extremely high. Previous studies of neonatal bacterial meningitis showed that high CSF protein levels, which with an optimal cutoff value of 1.88 g/L [28], were associated with poor prognosis [29–31]. The only reported case of *K. pneumoniae* meningitis who died mentioned a CSF protein of 2.34 g/L [12]. Another study into the clinical features of patients with adult bacterial meningitis showed the CSF protein levels in such patients with good or poor outcomes were 2.18 ± 1.47 g/L and 4.03 ± 4.19 g/L, respectively [32]. Additionally, white blood cells, immunoglobulins, and complements are normally absent in CSF, but the inflammatory response triggered by bacteria during meningitis results in increasing protein levels in the CSF which coincides with the intensity of the inflammatory response. Cytokines produced in inflammation may cause impairment of cell structure and organ function, thus increasing the risk of morbidity [28]. Overall then, the sky-high level of CSF protein in our patients may have foreshadowed their outcomes.

It is also noteworthy that all three patients came from Zhangjiakou city in Hebei Province. We suspect that the virulence of the *K. pneumoniae* species in this area may be particularly high. Five major virulence factors of *K. pneumoniae* are known to contribute to the pathogenesis of infection. These are the capsular serotype, hypermucoviscosity phenotype, lipopolysaccharide, siderophores, and pili. *K* [10, 33]. Based on previous study, we also know that severe manifestations such as comatose mental status, septic shock, and concomitant extra meningeal infections were more common in community- acquired *K. pneumoniae* meningitis patients compared with community-acquired *Streptococcus pneumoniae* meningitis, and the 28-day mortality (44.4% versus 10.7%; *P* = 0.001) and inhospitable mortality (51.9% versus 14.3%; P = 0.001) were much higher [9]. Besides the 100% mortality, all of the three patients had comatose mental status, septic shock, and live abscesses, the characters tallied with the reported study and may due to the high virulence of *K. pneumoniae.* What’s worse, multidrug-resistant and hypervirulent (MDR-HV) *K. pneumoniae* strains had spread in the community, 13 MDR-HV strains were identified from a total of 218 *K. pneumoniae* liver abscess episodes in a Taiwan Hospital [34]. Given the three cases in our study all occurred in one city in Hebei province, a future study might examine the particular virulence factors of this strain of *K. pneumoniae*.

Although liver abscess drainage has been recommended for a better clinical response [19] and hepatic resection is preferred for patients with Acute Physiology and Chronic Health Evaluation II (APACHE II) scores of ≥15 [28]. In our cases, liver abscesses were less than 5 cm, Percutaneous drainage for small liver abscess adjacent to the liver capsule can cause complications such as bleeding or peritonitis. The poor outcomes might have no business with the lack of liver abscess drainage.

In conclusion, *K. pneumoniae* meningitis is a rare cause of meningitis around the world. When patients with risk factors such as T2DM and alcoholic cirrhosis present to the emergency department with clinical features of meningitis, providers should maintain a high degree of suspicion for potential *K. pneumoniae*-related ILAS complicated by meningitis. Strict glycemic control, early drainage of liver abscesses and appropriate antibiotic application are all recommended for treating this condition. All three patients we received unfortunately died in short order, and it’s uncertain whether emergent liver abscess drainage would have helped at that point given the patients’ already high levels of CSF protein indicating severe inflammation. Potentially unknown virulence factors of *K. pneumoniae* in Zhangjiakou may also play a role in these patients’ poor outcomes. These cases were shared with the international medical community with the hope for timely diagnoses of *K. pneumoniae* meningitis in the future and to promote progress on learning more about this bacteria’s pathogenesis and treatment.

## Data Availability

The datasets used and analyzed during the current study are available from the corresponding author on reasonable request.

## Abbreviations

APACHE II: Acute Physiology and Chronic Health Evaluation II
BG: Blood glucose
CSF: Cerebrospinal fluid
CRP: C-reactive protein
CT: Computed tomography
DKA: Diabetic ketoacidosis
ED: Emergency Department
EICU: Emergency Department Intensive Care Unit
GCS: Glasgow coma scale
ILAS: Invasive liver abscess syndrome
*K. pneumoniae*: *Klebsiella pneumoniae* bacteria
magA: Mucoid-associated gene A
rmpA: Regulator of mucoid phenotype gene A
T2DM: Type 2 diabetes mellitus
